# Disruption of* Plasmodium falciparum* histidine-rich protein 2 may affect haem metabolism in the blood stage

**DOI:** 10.1186/s13071-020-04460-0

**Published:** 2020-12-09

**Authors:** Yingchao Yang, Tongke Tang, Bo Feng, Shanshan Li, Nan Hou, Xiao Ma, Lubin Jiang, Xiaofang Xin, Qijun Chen

**Affiliations:** 1 MOH Key Laboratory of Systems Biology of Pathogens, Institute of Pathogen Biology, Chinese Academy of Medical Sciences and Peking Union Medical College, Beijing, China; 2 Key Laboratory of the Ministry of Health for Research on Quality and Standardization of Biotech Products, National Institutes for Food and Drug Control, Beijing, China; 3 Key Laboratory of Zoonosis, Shenyang Agriculture University, Shenyang, China; 4 Unit of Human Parasite Molecular and Cell Biology, Key Laboratory of Molecular Virology and Immunology, Institute Pasteur of Shanghai, University of Chinese Academy of Sciences, Chinese Academy of Sciences, Shanghai, 200031 China; 5 Beijing Red Cross Blood Center, Beijing, China

**Keywords:** HRP2, Haemoglobin, Haem metabolism, Haem biosynthesis, RNA-seq

## Abstract

**Background:**

Haem is a key metabolic factor in the life cycle of the malaria parasite. In the blood stage, the parasite acquires host haemoglobin to generate amino acids for protein synthesis and the by-product haem for metabolic use. The malaria parasite can also synthesize haem *de novo* on its own. *Plasmodium falciparum*-specific histidine-rich protein 2 (*Pf*HRP2) has a haem-binding site to mediate the formation of haemozoin, a biocrystallized form of haem aggregates. Notably, the gene regulates the mechanism of haemoglobin-derived haem metabolism and the* de novo* haem biosynthetic pathway in the *Pfhrp2*-disrupted parasite line during the intraerythrocytic stages.

**Methods:**

The CRISPR/Cas9 system was used to disrupt the gene locus of *Pfhrp2*. DNA was extracted from the transgenic parasite, and PCR, Southern blotting and Western blotting were used to confirm the establishment of transgenic parasites. RNA-sequencing and comparative transcriptome analysis were performed to identify differences in gene expression between 3D7 and *Pfhrp2*^*-*^-3D7 parasites.

**Results:**

*Pfhrp2*^*-*^ transgenic parasites were successfully established by the CRISPR/Cas9 system. A total of 964, 1261, 3138, 1064, 2512 and 1778 differentially expressed genes (DEGs) were identified in the six comparison groups, respectively, with 373, 520, 1499, 353, 1253 and 742 of these DEGs upregulated and 591, 741, 1639, 711, 1259 and 1036 of them downregulated, respectively. Five DEGs related to haem metabolism and synthesis were identified in the comparison groups at six time points (0, 8, 16, 24, 32, and 40 h after merozoite invasion). The genes encoding delta-aminolevulinic acid synthetase and ferrochelatase, both related to haem biosynthesis, were found to be significantly upregulated in the comparison groups, and those encoding haem oxygenase, stromal-processing peptidase and porphobilinogen deaminase were found to be significantly downregulated. No GO terms were significantly enriched in haem-related processes (*Q* value = 1).

**Conclusion:**

Our data revealed changes in the transcriptome expression profile of the *Pfhrp2*^*-*^-3D7 parasite during the intraerythrocytic stages. The findings provide insight at the gene transcript level that will facilitate further research on and development of anti-malaria drugs.
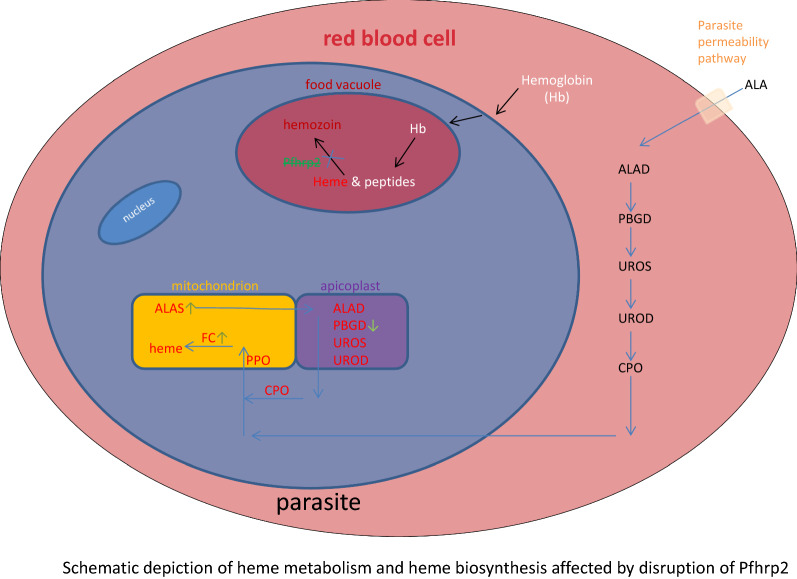

## Introduction

Malaria remains one of the most significant health challenges to human health. *Plasmodium falciparum* is one of the deadliest and most burdensome human malaria parasites, causing approximately 228 million cases of malaria, resulting in 405,000 deaths, worldwide in 2018 [1].

Haem is a crucial metabolic factor that is derived primarily from the parasite’s haem biosynthesis pathway [2] at the early ring stage and from haemoglobin digestion at later stages [3]. Malaria parasites ingest more than 75% of the host cell’s haemoglobin within a short period due to their nutritional requirements in the blood stage [3, 4]. The haemoglobin is digested in the cell’s food vacuole to generate amino acids, releasing the toxic haem moiety [5]. Since haem is toxic, excess haem is stored as the pigment haemozoin, a biocrystallized form of haem aggregates [6].

*Plasmodium falciparum*-specific histidine-rich protein 2 (*Pf*HRP2, PlasmoDB: PF3D7_0831800, https://www.plasmodb.org) constitutes two exons, and it is a water-soluble protein that is released from infected erythrocytes and circulated in malaria-infected patients [7, 8]. HRPII and HRPIII, as homologous proteins, can bind haem in the digestive vacuole and play a role in haemozoin formation [9–11]. Chloroquine binds to toxic haem metabolites, thereby preventing their conversion to and deposition as the inert haemozoin [12]. The malaria parasite also has a haem biosynthetic pathway to acquire haem. Studies with *Plasmodium berghei*-infected mice and *P. falciparum* in cultured delta-aminolevulinic acid synthetase (ALAS) and ferrochelatase (FC) knockout (KO) parasites have indicated that the haem biosynthetic pathway is nonessential for parasite survival in the blood stages [13, 14].

The clustered regularly interspaced short palindromic repeats/CRISPR-associated protein 9 (CRISPR/Cas9) system has been successfully used in genome editing of the human malaria parasites *P*. *falciparum* and *Plasmodium yoelii* [15–17]. Briefly, a single-guide RNA (sgRNA) guides the Cas9 endonuclease to cause double-strand breaks (DSBs), and DSBs can be repaired by homologous recombination using donor DNA. Transgenic parasites can be obtained after 3–6 weeks [15, 16]. This has been demonstrated to be a highly precise and efficient method for genome editing.

The aim of the study reported here was to specifically disrupt the gene locus of *Pfhrp2* in the wild-type 3D7 parasite and investigate how explicit gene disruption affects haemoglobin-derived haem metabolism and the haem biosynthetic pathway at the gene level.

## Methods

### Parasite culture, synchronization and pellet collection

*Plasmodium falciparum* (3D7 strain) cells at the asexual stage were cultured* in vitro* in human erythrocytes (blood group O+) obtained from the Beijing Red Cross Blood Center. The parasite was grown under 5% O_2_ and 5% CO_2_ in RPMI-1640 medium supplemented with 5 g/l Albumax II (Life Technologies, Carlsbad, CA, USA), 2 g/l sodium bicarbonate, 25 mM HEPES (pH 7.4, adjusted with KOH), 1 mM hypoxanthine and 50 mg/L gentamicin, as previously described [18].

For synchronization, parasite cells were cultured to at least 10% parasitaemia in T-75 flasks containing 50 ml of medium at 1% haematocrit. The cells were then transferred from the flask to a 50-ml tube and centrifuged for 5 min at 500 *g*, following which the supernatant was removed. The pellet was then suspended in 15 ml of 5% D-sorbitol solution and incubated at 37 °C for 10 min, follwed by centrifugation and removal of the supernatant. The culture was synchronized with three rounds of sorbitol treatment. The invasion culture solution was then collected at 8, 16, 24, 32, 40 and 46 h; three replicate samples were collected at each time point. A total of thirty-six samples were collected and centrifuged and the pellets stored at − 80 °C until use.

### Plasmid constructs and* Plasmodium* transfection

**Fig. 1:**
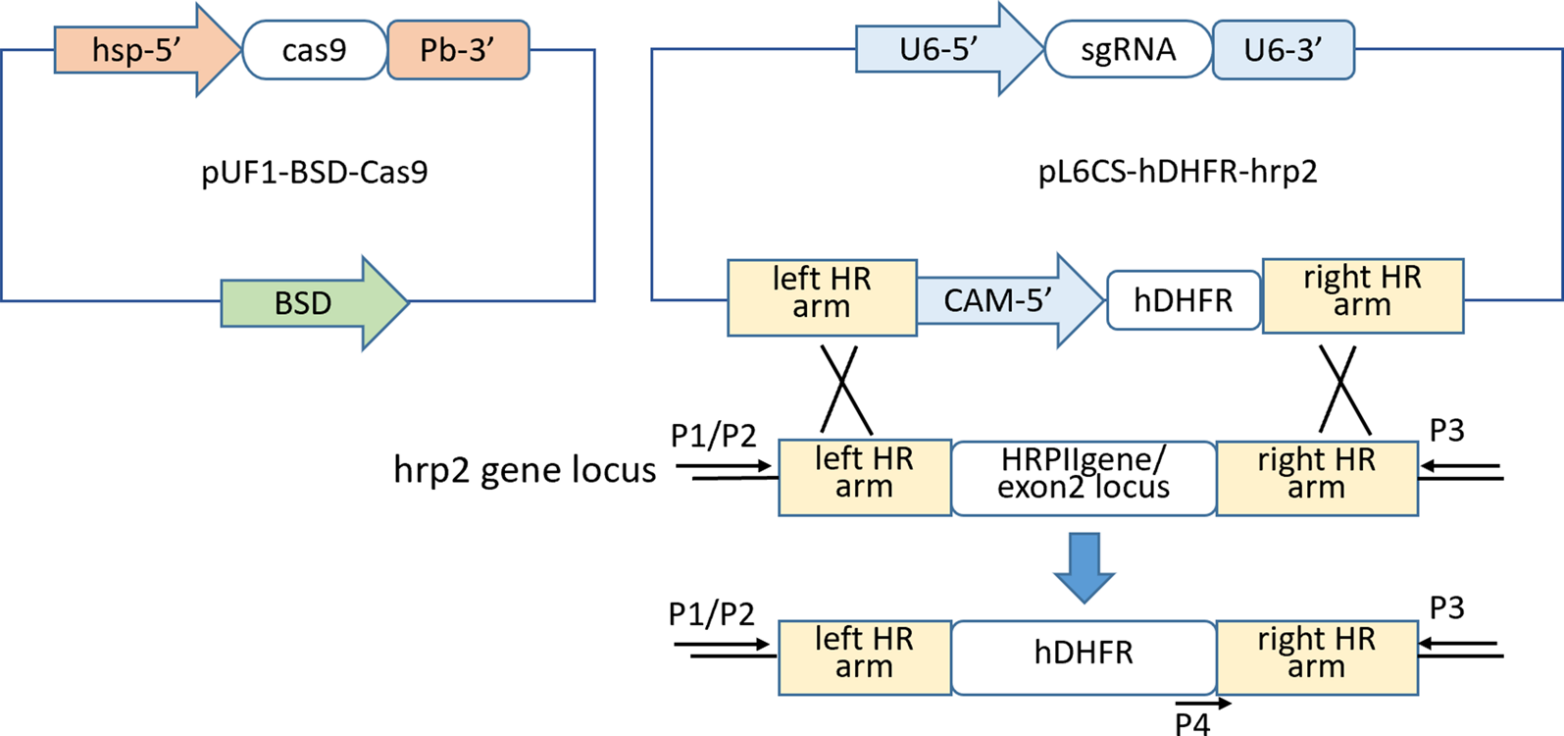
Schematic illustration of the underlying principle of the histidine-rich protein 2 (*hrp2*) gene deletion using the CRISPR-associated protein 9 (CRISPR/Cas9) system. The* hrp2* gene was replaced by human dihydrofolate reductase (*hDHFR*) sequences through homologous recombination that occurred at the left and right homologous arms (*left* and* right HR*, respectively). *BSD* Blasticidin S deaminase,* P1*,* P2* primers for PCR to check for hDHFR,* sgRNA* single-guide RNA

Using the pUF1-Cas9 and pL6CS plasmids kindly provided by Jose-Juan Lopez-Rubio, we constructed the pUF1-BSD-Cas9 and pL6CS-hDHFR-*hrp2* plasmids constructed to disrupt the *Pfhrp2* locus (Fig. 1). Construction of these two plasmids and *Plasmodium* transfection were performed as described previously [19].

Briefly, the pUF1-BSD-Cas9 plasmid expresses Cas9 nuclease and blasticidin S deaminase (BSD), while the pL6CS-hDHFR-*hrp2* plasmid expresses donor DNAs and sgRNAs targeting the *Pf*hrp2 gene (guide^hrp2^), and its left and right homologous arms were amplified separately by PCR from the genomic DNA of *P. falciparum* 3D7 (primers P1/P2 for the left arm and P3/P4 for right arm). The left homologous arm of* hrp2* was ligated between the ASCI and AfLII sites of the pL6CS plasmid. The right homologous arm was ligated between the HindIII and EcoRI sites of the plasmid. The right homologous arm was also used as a stop codon for the gene encoding hDHFR. The annealing reaction solution consisted of 1 μl of forward oligo (100 µM), 1 μl of reverse oligo (100 µM) and 23 μL of ddH_2_O. The annealing conditions for the sgRNA were as follows: 2 cycles of 30 s at 94 °C and 30 s at 72 °C, followed by 30 s at 60 °C, 30 s at 25 °C, and 30 s at 16 °C. The annealed sgRNA was then ligated into the pL6CS plasmid.

The transitional construct pL6CS-hDHFR-*hrp2* was transformed into competent cells, and plasmids were extracted and checked with restriction enzyme digestion and sequencing. After the correct transitional pL6CS-hDHFR-*hrp2* construct was obtained, this transitional plasmid was linearized with* Avr*II and *Xho*I. The sgRNAs of *hrp2* were annealed and inserted into the linearized transitional-pL6CS-hDHFR-*hrp2* plasmid using the In-Fusion kit (Takara Bio, Kusatsu, Shiga, Japan). The construct was transformed into competent cells once again and extracted. The final plasmid was verified by restriction enzyme digestion and DNA sequencing, following which it was isolated and used for electroporation to generate transgenic *P. falciparum* strains.

Electroporation of the two plasmids was carried out by the spontaneous uptake method using approximately 50 μg of maxi-prepped plasmid DNA and eight square wave electroporation pulses of 356 V for 1 ms each, separated by 0.1 s. Drugs (final concentration of 2.5 mg/l for blasticidin S and 5 nmol/l for WR99210) were added into the complete medium post-transfection to kill the parasites that lacked episomal pUF1-BSD-Cas9 and pL6CS-hDHFR-*hrp2*. The location of sgRNA in the *hrp2* and gene disruption schematic are listed in Additional file 1: Figure S1. All primer and sgRNA sequences used for constructing the plasmids are listed in Additional file 2: Table S1 and Additional file 3: Table S2.

### Confirmation of successful transgene introduction via PCR

**Fig. 2:**
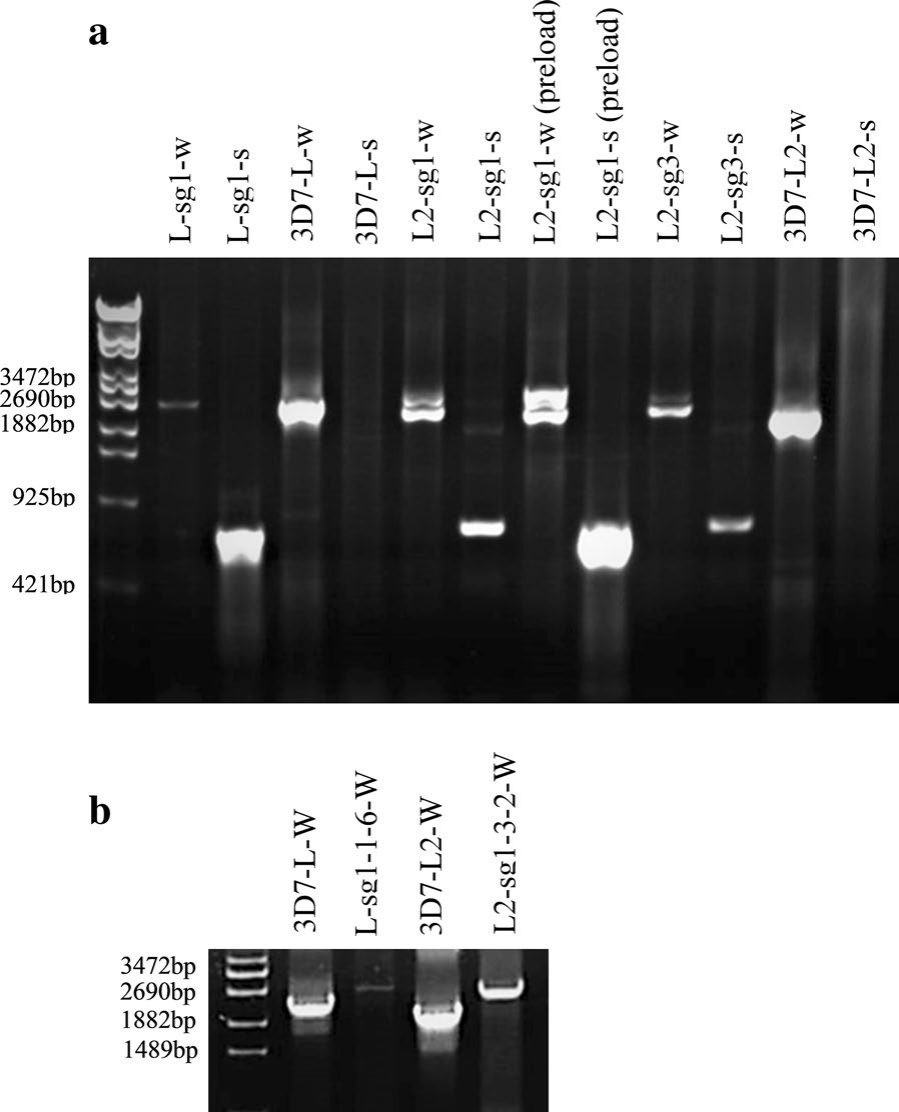
Genomic DNA PCR to confirm gene disruption. **a** Genomic DNA PCR to confirm the successful modification of the* Pfhrp2* gene by the sgRNA. **b** Genomic DNA PCR to verify *hrp2* deletion in monoclonal parasites.* 3D7* Wild-type strain of* Plasmodium falciparum*,* L*,* L2* transgenic parasites that were disrupted in exon 1 plus exon 2 and only exon 2 of* Pfhrp2*, respectively. PCR product sizes of the whole fragment (*w*) and partial fragment (*s*) were 2903 and 725 bp, respectively

Live *P. falciparum* cells were detected 20 days after electroporation, and genomic DNA was extracted from the harvested parasite pellets using the Qiagen DNA Extraction kit (Qiagen, Valencia, CA, USA). PCR was performed in a total volume of 20 μl, consisting of 10× buffer with 15 nM MgCl_2_, 200 μM dNTPs, 15 μM forward and reverse primers (P1/P2, as indicated in Fig. 2), 0.69 units of *Taq* DNA polymerase and 2 μl of DNA template. An* in vitro* *P. falciparum* 3D7 culture was used as a positive control for the *Pfhrp2* gene amplification experiments. All PCR products were separated and visualized on agarose gels, and products with the expected size were submitted for sequencing for further confirmation.

### Southern blotting analysis

Genomic DNA was isolated from transgenic parasites as described above. In total, 5 µg of the parasite genomic DNA was digested overnight using *Pst*I or *SacI* restriction endonuclease (Takara Bio). The DNA products were separated in a 1.0% agarose gel and transferred to a Hybond™–N^+^ membrane (Amersham™, GE Healthcare, Chicago, IL, USA) using the high-salt capillary transfer method. Probes were PCR amplified, cleaned, and labelled with DIG-dUTP using the PCR DIG Probe Synthesis kit (Roche Life Science, Penzberg, Germany). The blots were hybridized with the labelled probes, washed, exposed to film and visualized in a cassette. All primers used for southern blotting analysis are listed in Additional file 4: Table S3.

### Western blotting analysis

Successfully generated transgenic parasites were cultured in flasks. For the analysis of* Pfhrp2* expression, when parasitaemia exceeded 5%, infected red blood cells (iRBCs) were collected and incubated with 0.15% saponin lysis solution on ice for 7 min. After centrifugation at 500* g* for 5 min at room temperature (RT), the supernatants were collected and added to an appropriate amount of sodium dodecyl sulphate-polyacrylamide gel electrophoresis sample buffer, denatured at 95 °C for 8 min, resolved by electrophoresis in a 12.5% polyacrylamide gel (Life Technologies, Carlsbad, CA, USA) and transferred onto a 0.2-µm polyvinylidene difluoride (PVDF) membrane (Hybond LFP; GE Healthcare). HRP2 was specifically detected by using an anti-*Plasmodium falciparum* monoclonal antibody (MPFG-55P; horseradish peroxidaseP; Abcam, Oxford, UK).

### Total RNA extraction

Total RNA was extracted using TRIzol according to the manufacturer’s protocol. Briefly, pellets collected at different times from transgenic parasites were ground into powder in liquid nitrogen and transferred into a new tube with TRIzol reagent. The mix was shaken and kept for 5 min at RT and then centrifuged at 10,000 *g* for 5 min at 4 °C. Chloroform/isoamyl alcohol (24:1) was added to the supernatant with lysis reagents. After centrifugation at 10,000 *g* for 10 min at 4 °C, the supernatant was transferred into a new tube with an equal volume of isopropanol and kept at − 20 °C for 1 h. After centrifugation at 13,600 *g* for 20 min at 4 °C, the supernatant was precipitated by ethanol and dried for 3 min. The RNA pellet was dissolved with RNase-free water.

### mRNA library construction and sequencing

Oligo (dT)-attached magnetic beads were used to purify mRNA from parasite pellets. Purified mRNA was fragmented into small pieces with buffer at the appropriate temperature. First-strand cDNA was generated using random hexamer-primed reverse transcription, followed by second-strand cDNA synthesis. Then, A-tailing mix and RNA index adapters were added via incubation for end repair. The cDNA fragments were amplified by PCR, purified by Ampure XP Beads (Beckman Coulter Inc., Brea, CA, USA) and then dissolved in EB solution. The double-stranded PCR products were denatured by heating and circularized using the splint oligo sequence to obtain the final library. The single-strand circular DNA (ssCir DNA) was formatted as the final library. The final library was amplified with phi29 DNA polymerase to make DNA nanoballs (DNBs), which had more than 300 copies of one molecule. The DNBs were loaded into the patterned nanoarray, and paired-end 100-base reads were generated on the BGISEQ500 platform (BGI, Shenzhen, China).

### RNA sequencing data analysis

The RNA sequencing (RNA-Seq) data were filtered with SOAPnuke (v1.5.2) [20] by (1) removing reads containing sequencing adapters; (2) removing reads with low-quality base (base quality ≤ 5) ratios of > 20%; and (3) removing reads with unknown base (‘N’ base) ratios of > 5%. The clean reads were stored in the FASTQ format and mapped to the reference *P. falciparum* 3D7 genome (assembly GCA_000002765) using HISAT2 (v2.0.4) [21]. Bowtie2 (v2.2.5) [22] was applied to align the clean reads to the reference coding gene set, and then the gene expression levels were calculated by RSEM (v1.2.12) [23] and normalized to fragments per kilobase of transcript per million mapped reads (FPKM) values. Functional annotation of genes was achieved by mapping genes to the Gene Ontology (GO, https://www.geneontology.org) and Kyoto Encyclopedia of Genes and Genomes (KEGG, https://www.genome.jp/kegg/) databases using BLAST software (V2.2.23). GO annotation was performed by Blast2GO (v 2.5.0) with NR annotations. DESeq2 (v1.4.5) [24] was used to detect differentially expressed genes (DEGs), and DEGs with fold change of > 2 or < − 2 and adjusted *P* value ≤ 0.001 were considered to be significant DEGs. GO enrichment analysis was performed using Phyper (https://en.wikipedia.org/wiki/Hypergeometric_distribution), a function of R (R Foundation for Statistical Computing, Vienna, Austria). The significant levels of terms and pathways were corrected by the* Q* value with a rigorous threshold (*Q* ≤ 0.05).

## Results

### Successful establishment of HRP2-kO* Plasmodium falciparum*

The pUF1-BSD-Cas9 plasmid was constructed from the pUF1-Cas9 plasmid. The *P. falciparum* 3D7 strain was then transfected with 50 µg of pL6cs-hDHFR-*hrp2* (donor DNA) and 50 µg of pUF1-BSD-Cas9 via electroporation. For selection of the successfully transfected parasite, the drugs BSD and WR99210 were added to the culture medium 1 day after electroporation.

Approximately 20 days after electroporation, live parasites could be seen in the culture under selection with BSD and WR99210. A portion of the live parasite population was collected for genomic DNA isolation, and PCR was performed to validate the modification of the *Pfhrp2* gene. In these PCR assays, two primers were designed for the genomic DNA sequences beyond the left and right homologous arms (P1/P2; Fig. 2) to prevent contamination from the episomal plasmid template. PCR products were analysed by agarose electrophoresis and sequenced for confirmation.

**Fig. 3:**
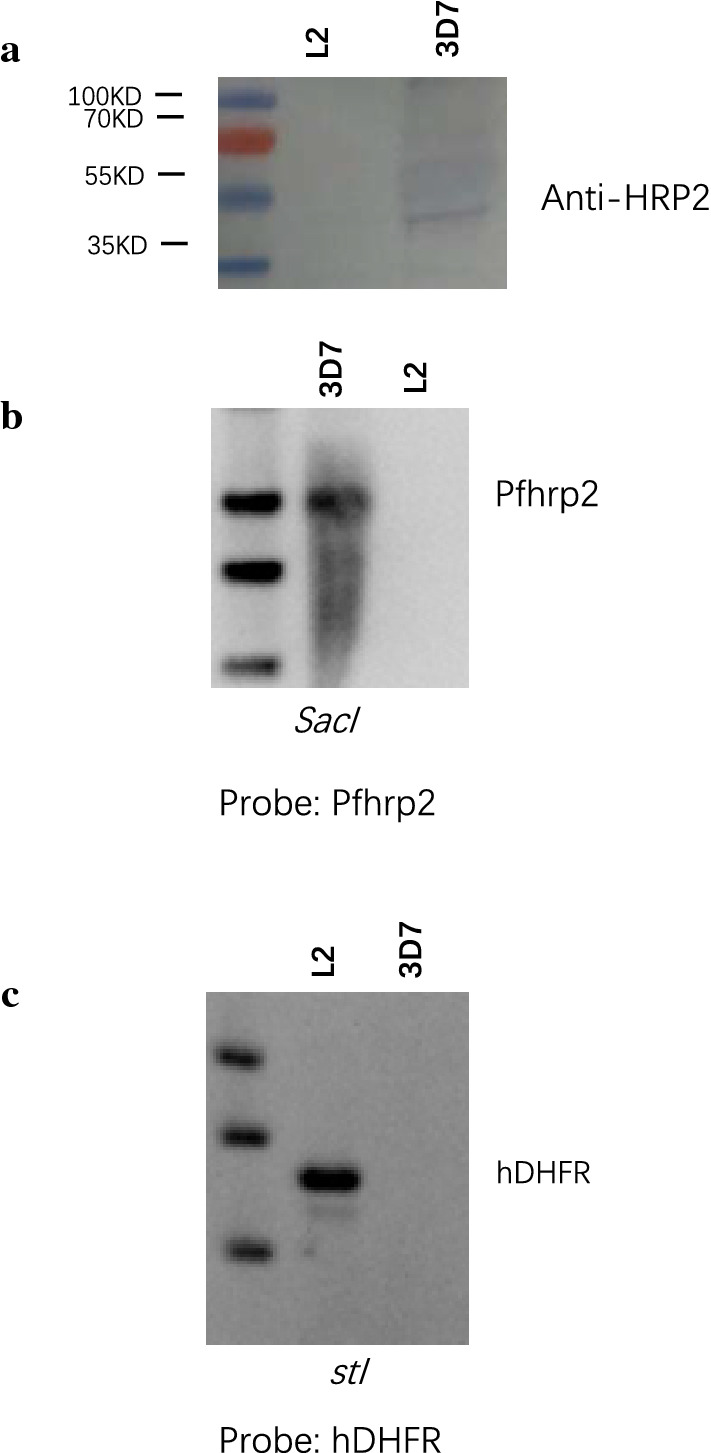
**a** Western blotting analysis to confirm the expression of the* Pf*HRP2 protein. The supernatants of the parasite culture medium were separated by sodium dodecyl sulphate-polyacrylamide gel electrophoresis, and a mouse monoclonal (MPFG-55P) antibody against* Plasmodium falciparum* (horseradish peroxidase [HRP]) was the primary antibody used for the Western blotting analysis to confirm HRP2 protein expression. HRP–goat anti-mouse was the secondary antibody. **b** Southern blotting analysis to confirm the disruption of* Pf*hrp2. The genomic DNA was digested overnight using the* Sac*I restriction endonuclease. The DNAs were separated in an agarose gel and transferred to a membrane. The blots were hybridized with the labelled* Pf*hrp2 probe and exposed for 10 min. **c** Southern blotting analysis to confirm the presence of the drug resistance gene hDHFR. The genomic DNA was digested overnight using the* Pst*I restriction endonuclease. The DNAs were separated in an agarose gel and transferred to a membrane. The blots were hybridized with the labelled hDHFR probe and exposed for 40 min.* L2* Transgenic parasite, which was disrupted in only exon 2 of* Pfhrp2*

Transgenic parasites that had been checked by PCR and DNA sequencing were further checked by Southern blotting analysis. Two experiments were performed to verify that the *Pfhrp2* gene had been replaced by the drug resistance gene hDHFR. One experiment was performed to indicate that the wild-type strain 3D7 still possessed the *Pfhrp2* gene while the transgenic parasite did not (Fig. 3b). The other experiment was conducted to prove that the *Pfhrp2* gene was replaced by the hDHFR gene (Fig. 3c). Southern blotting analysis results showed that only the hDHFR gene was detected in the transgenic parasite. Therefore, we concluded that the *Pfhrp2* gene had been successfully knocked out from the *P. falciparum* 3D7 strain using the CRISPR/Cas9 system.

Transgenic parasites that had been checked by PCR, DNA sequencing and Southern blotting analysis were further confirmed by western blotting analysis. The original molecular weight of the protein was 32.41 kD (Fig. 3a). Western blotting results showed that the molecular weight of HRP2 was not the same as its theoretical molecular weight. The band corresponding to HRP2 appeared to be slightly larger than its theoretical molecular weight, which may be a result of post-translational modification.

Based on these results, we concluded that the CRISPR/Cas9 system had been successfully used to knock out the* Pfhrp2* gene.

### Overview of the RNA-Seq data

A total of 36 samples were tested using the BGISEQ-500 platform, and at least 6.0 Gb of clean data from each sample were obtained from transcriptome sequencing and were available for further expression level analysis after quality control. Total clean reads from the RNA-Seq data were mapped uniquely to the 3D7 genome. More than 97% of the total clean reads had Phred-like quality scores at the Q20 level (Additional file 5: Table S4).

The average comparison rate of the sample genome was 57.39%, and the average comparison rate of the comparison gene set was 49.26%. A total of 5427 expressed genes were detected, of which 5350 were known genes; 77 new genes were predicted (Additional file 6: Table S5). A total of 2358 new transcripts were detected, of which 2078 belonged to new alternative splicing subtypes of known protein-coding genes and 77 belonged to new protein-coding gene transcripts; the remaining 203 transcripts were long non-coding RNAs. Length distribution of the transcripts is listed in Additional file 7: Table S6. Via comparative transcriptome analysis, these RNA-Seq data provided a solid foundation for identifying the genes participating in biological processes.

### Analyses of differentially expressed genes

**Fig. 4:**
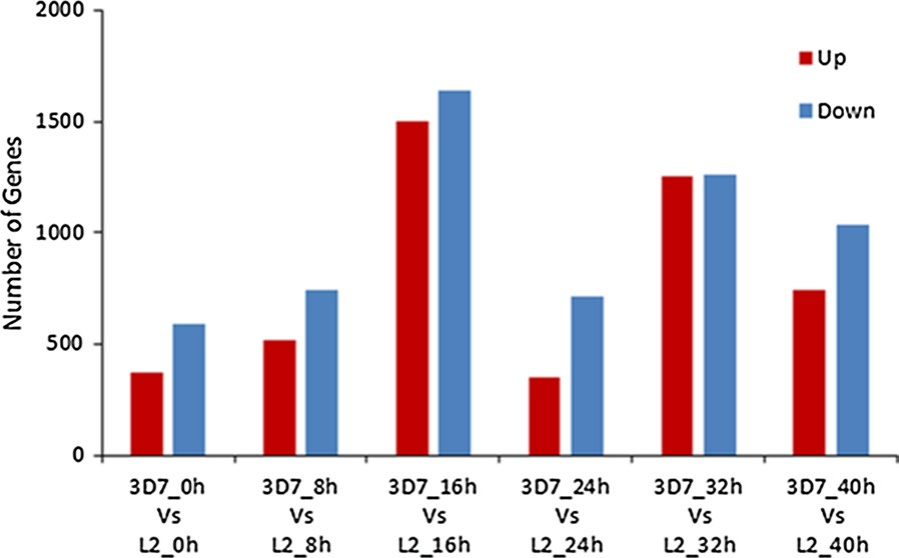
Statistics of differentially expressed genes and gene regulation in the comparison groups.* Up*,* down* Up-, downregulated, respectively

To investigate changes in gene expression profiles, DESeq2 was used to detect DEGs, and DEGs with a fold change of > 2 or < − 2 and adjusted *P* value of ≤ 0.001 were considered to be significant DEGs. Differential expression analysis identified 964, 1261, 3138, 1064, 2512 and 1778 DEGs in the comparison groups 3D7_0h* vs* L2_0h, 3D7_8h* vs* L2_8h, 3D7_16h* vs* L2_16h, 3D7_24h* vs* L2_24h, 3D7_32h* vs* L2_32h and 3D7_40h* vs* L2_40h, respectively. Differential expression analysis for the comparison groups 3D7_0h* vs* L2_0h, 3D7_8h* vs* L2_8h, 3D7_16h* vs* L2_16h, 3D7_24h* vs* L2_24h, 3D7_32h* vs* L2_32h and 3D7_40h* vs* L2_40h identified 373, 520, 1499, 353, 1253 and 742 upregulated genes, respectively, and 591, 741, 1639, 711, 1259 and 1036 downregulated genes, respectively (Fig. 4). The DEGs identified in the comparison groups can help us understand the gene regulatory mechanisms underlying the parasite response to KO of the* Pfhrp2* gene.

**Fig. 5:**
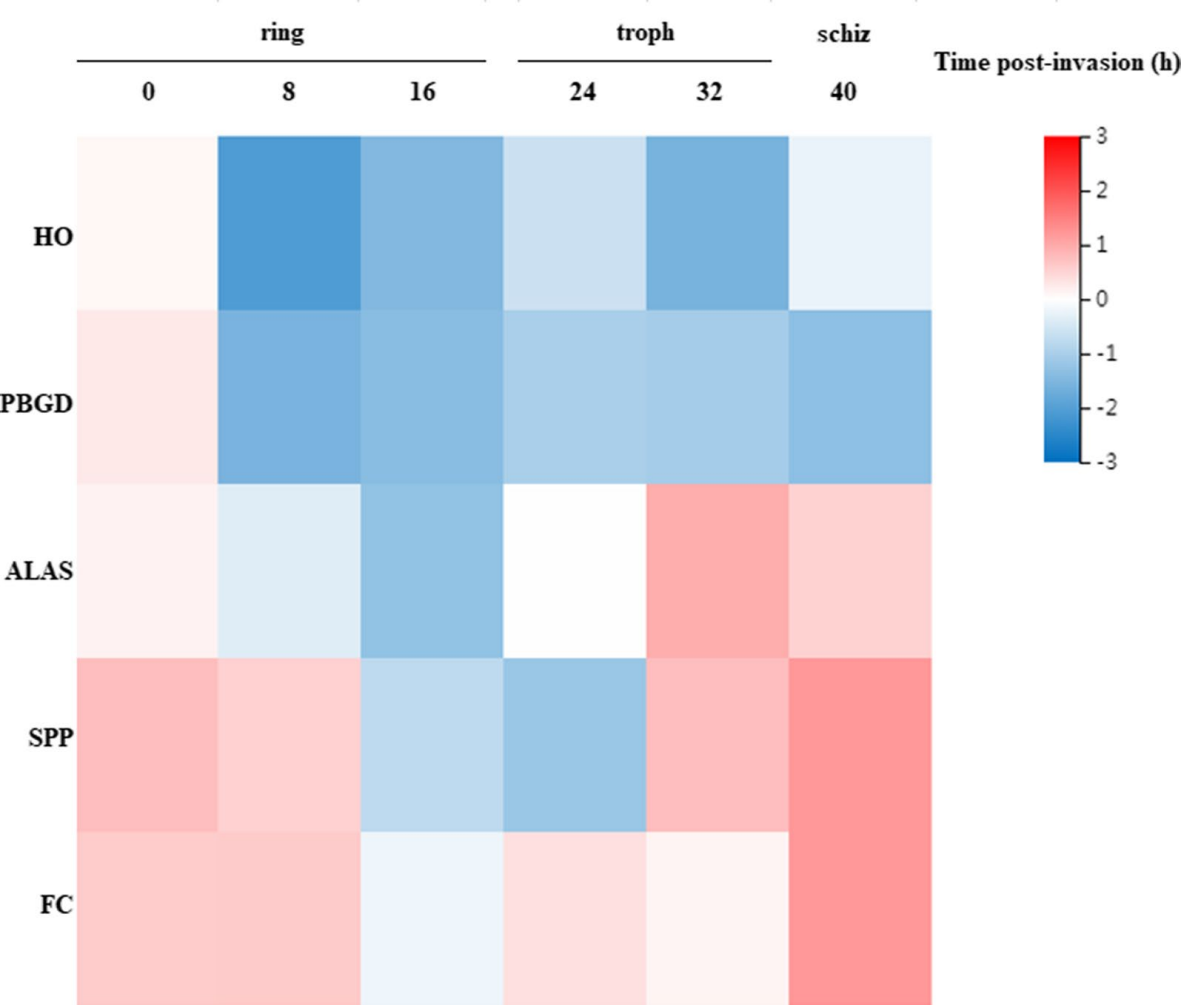
Transcription pattern of the genes involved in haem metabolism. Heat map representation of the relative transcription activity of five genes at six time points during asexual erythrocytic growth. The horizontal axis represents the expression level of each gene calculated as Log2(FPKM + 1). The values range from − 3 (lowest, blue) to + 3 (highest, red).* HO* Haem oxygenase (PlasmoDB:PF3D7_1011900),* PBGD* porphobilinogen deaminase (PlasmoDB:PF3D7_1209600),* ALAS* delta-aminolevulinic acid synthetase (PlasmoDB:PF3D7_1246100),* SPP* stromal-processing peptidase (PlasmoDB:PF3D7_1440200),* FC* ferrochelatase (PlasmoDB:PF3D7_1364900)

A total of five DEGs related to haem metabolism and synthesis were identified (Additional file 8: Table S7), including genes encoding ALAS (PlasmoDB:PF3D7_1246100), FC (PlasmoDB:PF3D7_1364900), haem oxygenase (HO, PlasmoDB:PF3D7_1011900), stromal-processing peptidase (SPP, PlasmoDB:PF3D7_1440200) and porphobilinogen deaminase (PBGD, PlasmoDB:PF3D7_1209600) (Fig. 5). The ALAS and FC genes were found to be significantly upregulated in the comparison groups, while the HO, SPP and PBGD genes were found to be significantly downregulated in the comparison groups.

### Enrichment analysis of GO terms

Functional annotation and classification were performed by comparing the sequences with the GO database. Enrichment analyses of GO terms were performed for DEGs using the phyper function in R software. The* P* values were first calculated, and then the false discovery rate correction was performed on the* P* values. In general, a function with a* Q* value ≤ 0.05 is regarded as being significantly enriched. As* Pf*HRP2 mediates haemozoin formation and is involved in haem-related processes, DEGs in the comparison groups were primarily associated with GO terms related to haem metabolism processes, such as HO (decyclization) activity and haem biosynthetic processes. However, no GO terms were significantly enriched in the above-mentioned haem-related process (*Q* value = 1).

## Discussion

The traditional method to edit *P. falciparum* genes is very inefficient and requires several months for knock-in/out of target genes. This greatly limits molecular studies on malaria parasites. Recently, CRISPR/Cas9 has been used for gene editing in various organisms, including *Plasmodium* species [15, 16, 25].

Our CRISPR/Cas9 system contains homologous arms (donor DNA fragments), sgRNA and a selectable marker in one plasmid and Cas9 nuclease with a selectable marker in another plasmid. Twenty days after electroporation, specific gene-disrupted parasites were detected. In this study, we successfully applied this CRISPR/Cas9-based genome editing system to disrupt the *Pfhrp2* gene of *P. falciparum* strain 3D7.

We observed that the growth of the parasite in the host was not affected by the deletion of *Pfhrp2* compared with the growth of the wild type. In support of this finding, HRP2 gene deletion mutants of *P. falciparum* of multiple genetic origins have been reported in South America, Asia and Africa [26–29]. These results all prove that *Pfhrp2* is not an essential gene for the survival of parasites during intraerythrocytic stages.

Malaria parasites have haemoglobin-derived haem metabolism and *de novo* haem biosynthesis pathways. During the intraerythrocytic stages, parasites ingest host cell haemoglobin within the food vacuole to supply amino acids for growth and release toxic haem. The released by-product haem can bind with HRP2 and HRP3 to become haemozoin [12]. HO may enzymatically degrade some haem to biliverdin or its downstream metabolite bilirubin [30].

According to our RNA-Seq data, with disruption of *Pfhrp2,* the transcript level of *PfHO* was significantly downregulated, which further affected haem metabolism. At the same time, the transcripts levels of genes encoding enzymes related to the *de novo* haem biosynthesis pathway in the mitochondrion of *P. falciparum* 3D7, such as ALAS (the first enzyme of the pathway) and FC, were upregulated to increase haem supply to the parasite. However, the transcript expression of PBGD, which catalyses the conversion of porphobilinogen to hydroxymethylbilane in the apicoplast of the parasite, was downregulated, possibly decreasing haem biosynthesis in the apicoplast of the parasite.

*Plasmodium falciparum* contains a vestigial, non-photosynthetic plastid—the apicoplast. Numerous proteins encoded by nuclear genes are targeted to the apicoplast because of N-terminal extensions. The first part of this leader sequence is a signal peptide that targets proteins to the secretory pathway. The second so-called transit peptide region is required to direct proteins from the secretory pathway across the multiple membranes surrounding the apicoplast. The transit peptide of apicoplast-targeted proteins are cleaved and then imported into the apicoplast. SPP performs this cleavage reaction. SPP also shares a leader sequence with ALAD via an alternative splicing event [31–33]. ALAD is also involved in the haem biosynthesis pathway. The SPP gene was significantly downregulated in the comparison groups, which would result in a decrease the amount of protein imported into the apicoplast and ultimately affect haem biosynthesis in the apicoplast.

When *Pfhrp2* was disrupted, haem metabolism in the food vacuole and haem biosynthesis in the apicoplast were influenced, and the gene transcript levels of the enzymes participating in haem biosynthesis in the mitochondrion of the parasite were upregulated. It has been reported that vestigial host enzymes within the erythrocyte cytoplasm can replace the haem biosynthesis process in the apicoplast [34]. For this reason, *de novo* haem biosynthesis can reduce the influence of the downregulation of PBGD and SPP. Combined with the observation that the growth of parasites did not change, our results indicate that the haem level in the parasite remained stable. Studies utilizing liquid chromatography-tandem mass spectrometry and metabolomics strategies should be conducted to confirm this in the near future.

It has been reported that the haem biosynthesis pathway is nonessential in the blood stage because ALAS and FC gene KO did not affect parasite survival. This conclusion is inappropriate because parasites can acquire haem from two pathways for survival in the blood stage. When one pathway encounters a problem, the other pathway provides haem for the parasite. Our research proved that haem metabolism and the haem biosynthesis pathway are both needed at the gene transcript level in the blood stage for parasite survival.

Chloroquine was discovered and derived from quinine in 1934 [35]. It is effective against the malarial parasite during its intraerythrocytic stages and can inhibit the HRP-mediated synthesis of haemozoin and disrupt the haem pathway that occurs within the acidic digestive vacuole of the parasite [12]. Our research has shown that intraerythrocytic-stage parasites not only possess a haem biosynthesis pathway but also can use host enzymes and substances to assist in haem biosynthesis. The FC KO parasite cells grew normally and exhibited no changes in sensitivity to haem-related antimalarial drugs [36]. Parasites can acquire haem from two different pathways, and disruption of only *Pfhrp2* or enzymes of haem biosynthesis cannot exterminate the haem supplement completely during the intraerythrocytic stages. Therefore, haem metabolism and the haem biosynthesis pathway are not viable targets for traditional drug inhibition [13, 36]. A cooperative mechanism exists between the haem biosynthesis and metabolism pathways for parasite growth and survival in the blood stage. It may be possible to disrupt haem supplementation by simultaneous suppression of haem metabolism and the haem biosynthesis pathway. We believe that our work will be beneficial for understanding haem acquisition and drug resistance during intraerythrocytic stages.

## Conclusion

The results suggest that disruption of *Pfhrp2* alters the parasite’s haem metabolic and biosynthesis pathways at the gene transcript level. A cooperative mechanism exists between the haem biosynthesis and metabolic pathways for parasite growth and survival in the blood stage. It is difficult to treat malaria patients by inhibiting only one pathway with traditional antimalarial drugs.

## Supplementary information

**Additional file 1: Figure S1.** HRPII gene and gene disruption schematic.

**Additional file 2: Table S1.** Primer sequences used for sgRNA synthesis.

**Additional file 3: Table S2.** Primer sequences used for donor construction and the detection of hDHFR gene identification.

**Additional file 4: Table S3.** Primer sequences used for Southern blot.

**Additional file 5: Table S4.** Overview of the RNA-Seq data.

**Additional file 6: Table S5.** The annotation of novel transcripts/genes.

**Additional file 7: Table S6.** Length distribution of transcripts.

**Additional file 8: Table S7.** Gene expression of five differentially expressed genes at six time points.

## Data Availability

The datasets generated and/or analysed during the current study are available under NCBI project PRJNA663197 (https://www.ncbi.nlm.nih.gov/bioproject/663197) with accession numbers for 72 objects (SAMN16122358~SAMN16122429). Any reasonable requests should be made to the corresponding author.

## Abbreviations

BSD: Blasticidin S deaminase
DSB: Double-strand break
hDHFR: Human dihydrofolate reductase
sgRNA: Single-guide RNA
