# Supplementary material for: Disruption of Plasmodium falciparum histidine-rich protein 2 may affect haem metabolism in the blood stage
Source: Parasit Vectors. 2020 Dec 9;13:611. doi: 10.1186/s13071-020-04460-0 (PMC7725123; doi:10.1186/s13071-020-04460-0)
Supplement: Supplementary file 1 — Additional file 1: Figure S1. HRPII gene and gene disruption schematic. [file 13071_2020_4460_MOESM1_ESM.docx]

**Table S1 Primer sequences used for sgRNA synthesis**

| Primer | Sequence (5’-3’) |
| --- | --- |
| HRPII sgRNA1 Forward | TAAGTATATAATATTGgtggcggcttcgtggtgtggttttagagctagaa |
| HRPII sgRNA1 Reverse | ttctagctctaaaaccacaccacgaagccgccacCAATATTATATACTTA |
| HRPII sgRNA2 Forward | TAAGTATATAATATTCcacaagttattattaaatggttttagagctagaa |
| HRPII sgRNA2 Reverse | ttctagctctaaaaccatttaataataacttgtgGAATATTATATACTTA |
| HRPII sgRNA3 Forward | TAAGTATATAATATTcgtgatgggcatcggctgcagttttagagctagaa |
| HRPII sgRNA3 Reverse | ttctagctctaaaactgcagccgatgcccatcacgAATATTATATACTTA |
