# Supplementary material for: Disruption of Plasmodium falciparum histidine-rich protein 2 may affect haem metabolism in the blood stage
Source: Parasit Vectors. 2020 Dec 9;13:611. doi: 10.1186/s13071-020-04460-0 (PMC7725123; doi:10.1186/s13071-020-04460-0)
Supplement: Supplementary file 2 — Additional file 2: Table S1. Primer sequences used for sgRNA synthesis. [file 13071_2020_4460_MOESM2_ESM.docx]

**Table S2. Primer sequences used for donor construction and**

**the detection of hDHFR gene identification**

| Primer | Sequence (5’-3’) |
| --- | --- |
| HRPII left arm HR Forward | GCGGCCCTAGTCTAGGGCGCGCCaaatttatcatacttctgtattttaaatc |
| HRPII left arm HR Reverse | TTTTTTTACAAAATGCTTAAGaatatagaatactaattttttgcataac |
| HRPII right arm HR Forward | GCGGCCCTAGTCTAGGGCGCGCCccctttttatataattaaaagtatagaac |
| HRPII right arm HR Reverse | TTTTTTTACAAAATGCTTAAGctatataaaaataaaaaagcatttaaaaatg |
| P1 | aaacaaaattaaattattataatccctac |
| P2 | ttaagtttctccatttttaatacttc |
| P3 | ttgcaaatatataataatatatcaagag |
| P4 | ctctgctgagaactaaattaattc |
