# Supplementary material for: Disruption of Plasmodium falciparum histidine-rich protein 2 may affect haem metabolism in the blood stage
Source: Parasit Vectors. 2020 Dec 9;13:611. doi: 10.1186/s13071-020-04460-0 (PMC7725123; doi:10.1186/s13071-020-04460-0)
Supplement: Supplementary file 3 — Additional file 3: Table S2. Primer sequences used for donor construction and the detection of hDHFR gene identification. [file 13071_2020_4460_MOESM3_ESM.docx]

**Table S3. Primer sequences used for Southern blot**

| Primer | Sequence (5’-3’) |
| --- | --- |
| hDHFR Forward | TGTGTAGCAAAAATGCAAAAGG |
| hDHFR Reverse | TTAATGGCGTAGGCAATGTG |
| Pfhrp2 Forward | TGGTGATTATGGGTAAGAAGAC |
| Pfhrp2 Reverse | ATAGTTTAAGATGGCCTGGGTG |
