# Supplementary material for: Disruption of Plasmodium falciparum histidine-rich protein 2 may affect haem metabolism in the blood stage
Source: Parasit Vectors. 2020 Dec 9;13:611. doi: 10.1186/s13071-020-04460-0 (PMC7725123; doi:10.1186/s13071-020-04460-0)
Supplement: Supplementary file 4 — Additional file 4: Table S3. Primer sequences used for Southern blot. [file 13071_2020_4460_MOESM4_ESM.docx]

**Table S4.** Overview of the RNA-Seq data

| **Sample** | **Raw reads number (M)** | **Clean reads number (M)** | **Total mapped reads (%)** | **Unique match (%)** | **Multi-position match (%)^a^** | **Q20 (%)^b^** |
| --- | --- | --- | --- | --- | --- | --- |
| 3D7_1_0h | 124.94 | 111.36 | 82.85 | 70.03 | 12.82 | 98.02 |
| 3D7_1_8h | 127.44 | 108.28 | 32.19 | 27.29 | 4.90 | 98.19 |
| 3D7_1_16h | 127.44 | 109.84 | 33.33 | 26.37 | 6.96 | 97.92 |
| 3D7_1_24h | 122.44 | 106.05 | 51.77 | 42.70 | 9.07 | 98.23 |
| 3D7_1_32h | 124.94 | 108.97 | 48.66 | 38.46 | 10.20 | 97.87 |
| 3D7_1_40h | 124.94 | 109.95 | 64.36 | 52.99 | 11.37 | 98.11 |
| 3D7_2_0h | 122.44 | 107.87 | 71.42 | 57.81 | 13.61 | 97.87 |
| 3D7_2_8h | 127.44 | 107.50 | 7.56 | 5.21 | 2.35 | 97.61 |
| 3D7_2_16h | 124.94 | 107.03 | 31.5 | 25.77 | 5.73 | 98.22 |
| 3D7_2_24h | 127.44 | 109.84 | 52.87 | 44.22 | 8.65 | 97.98 |
| 3D7_2_32h | 124.94 | 110.11 | 53.40 | 44.65 | 8.75 | 98.08 |
| 3D7_2_40h | 124.94 | 108.77 | 50.49 | 40.40 | 10.09 | 98.06 |
| 3D7_3_0h | 122.44 | 107.04 | 66.89 | 53.84 | 13.05 | 97.79 |
| 3D7_3_8h | 127.44 | 109.97 | 27.39 | 22.33 | 5.06 | 97.91 |
| 3D7_3_16h | 122.44 | 107.77 | 33.61 | 27.38 | 6.23 | 98.19 |
| 3D7_3_24h | 124.94 | 108.88 | 39.56 | 32.37 | 7.19 | 97.96 |
| 3D7_3_32h | 124.94 | 108.90 | 42.11 | 33.82 | 8.29 | 98.11 |
| 3D7_3_40h | 124.94 | 110.02 | 76.68 | 65.09 | 11.59 | 98.12 |
| L2_1_0h | 124.94 | 108.68 | 77.80 | 63.78 | 14.02 | 97.98 |
| L2_1_8h | 127.44 | 108.21 | 74.44 | 63.51 | 10.93 | 98.19 |
| L2_1_16h | 124.94 | 108.95 | 47.16 | 38.25 | 8.91 | 97.93 |
| L2_1_24h | 122.44 | 107.88 | 57.40 | 47.51 | 9.89 | 98.04 |
| L2_1_32h | 124.94 | 111.27 | 66.38 | 55.02 | 11.36 | 97.96 |
| L2_1_40h | 118.37 | 105.24 | 72.43 | 60.55 | 11.88 | 98.20 |
| L2_2_0h | 124.94 | 111.04 | 85.86 | 72.07 | 13.79 | 97.98 |
| L2_2_8h | 122.30 | 106.36 | 81.32 | 69.08 | 12.24 | 98.28 |
| L2_2_16h | 122.36 | 108.07 | 42.89 | 35.06 | 7.83 | 98.00 |
| L2_2_24h | 124.94 | 109.94 | 58.99 | 49.21 | 9.78 | 98.06 |
| L2_2_32h | 123.30 | 109.55 | 68.54 | 57.33 | 11.21 | 98.28 |
| L2_2_40h | 119.94 | 105.32 | 77.91 | 64.98 | 12.93 | 98.12 |
| L2_3_0h | 124.94 | 111.43 | 73.53 | 59.51 | 14.02 | 98.03 |
| L2_3_8h | 122.44 | 107.06 | 80.73 | 66.84 | 13.89 | 98.01 |
| L2_3_16h | 124.94 | 109.33 | 39.39 | 31.11 | 8.28 | 97.84 |
| L2_3_24h | 122.44 | 107.85 | 55.25 | 44.23 | 11.02 | 98.05 |
| L2_3_32h | 124.94 | 110.66 | 63.54 | 52.49 | 11.05 | 97.92 |
| L2_3_40h | 122.44 | 106.86 | 75.78 | 64.94 | 10.84 | 98.33 |

Notes

L2: the name of the transgenic parasite in which *Pfhrp2* had been knocked down.

^a^Total Mapped Reads (%) =Unique Match (%)+ Multi-position Match (%),the percentages of clean reads aligned to the reference genome.

^b^Q20 (%) is the percentage of reads with Phred quality scores over 20.
