# Supplementary material for: Disruption of Plasmodium falciparum histidine-rich protein 2 may affect haem metabolism in the blood stage
Source: Parasit Vectors. 2020 Dec 9;13:611. doi: 10.1186/s13071-020-04460-0 (PMC7725123; doi:10.1186/s13071-020-04460-0)
Supplement: Supplementary file 5 — Additional file 5: Table S4. Overview of the RNA-Seq data. [file 13071_2020_4460_MOESM5_ESM.docx]

**Table S****5.** The annotation of novel transcripts/genes

| Total Novel Transcript | Coding Transcript | Noncoding Transcript | Novel Isoform | Novel Gene |
| --- | --- | --- | --- | --- |
| 2358 | 2155 | 203 | 2078 | 77 |

StringTie was used to reconstruct the transcript of each sample, then use Cuffmerge to integrate the reconstruction information of all samples, and then use Cuffcompare to compare the integrated transcript with the reference annotation information, and select the class code type as ‘u’, ‘i’, ‘o’ ,‘j’ transcripts are defined as new transcripts.
