# Supplementary material for: Disruption of Plasmodium falciparum histidine-rich protein 2 may affect haem metabolism in the blood stage
Source: Parasit Vectors. 2020 Dec 9;13:611. doi: 10.1186/s13071-020-04460-0 (PMC7725123; doi:10.1186/s13071-020-04460-0)
Supplement: Supplementary file 6 — Additional file 6: Table S5. The annotation of novel transcripts/genes. [file 13071_2020_4460_MOESM6_ESM.docx]

**Table S6.** Length distribution of transcripts

| Length | Number |
| --- | --- |
| 0~300 | 162 |
| 301~500 | 411 |
| 501~700 | 468 |
| 701~900 | 491 |
| 901~1100 | 530 |
| 1101~1300 | 401 |
| 1301~1500 | 323 |
| 1501~1700 | 239 |
| total | 3025 |
