# Supplementary figures and images for: Disruption of Plasmodium falciparum histidine-rich protein 2 may affect haem metabolism in the blood stage
Source: Parasit Vectors. 2020 Dec 9;13:611. doi: 10.1186/s13071-020-04460-0 (PMC7725123; doi:10.1186/s13071-020-04460-0)

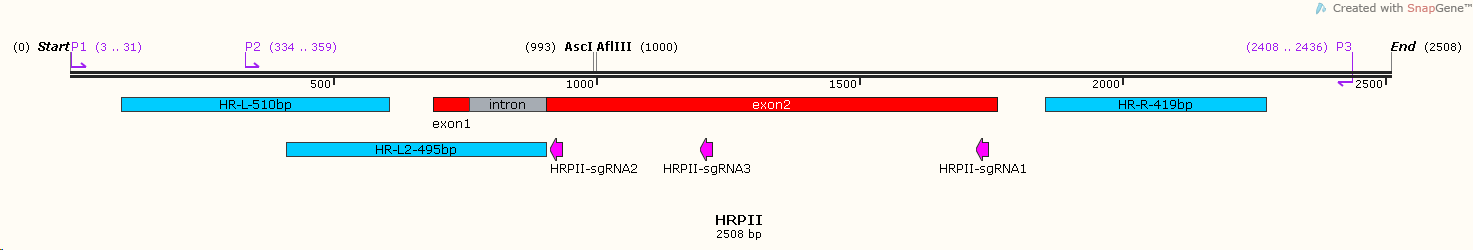
 **Figure S1.** HRPII gene and gene disruption schematic

Supplement: Supplementary file 8 — Additional file 8: Table S7. Gene expression of five differentially expressed genes at six time points. [file 13071_2020_4460_MOESM8_ESM.docx]
